# Complete Genome Sequence of *Lactiplantibacillus plantarum* Strain DM083 Isolated from Human Tongue Coating

**DOI:** 10.1128/mra.00675-22

**Published:** 2022-09-27

**Authors:** Do-Young Park, Jiyoung Hwang, Yunji Kim, Young-Youn Kim, Hye-Sung Kim, Inseong Hwang

**Affiliations:** a DOCSmedi Co., Ltd., Goyang-si, South Korea; b Apple Tree Institute of Biomedical Science, Apple Tree Medical Foundation, Goyang-si, South Korea; University of Maryland School of Medicine

## Abstract

We isolated Lactiplantibacillus plantarum DM083 from the human tongue coating to establish a strain library for oral probiotics. It has a single circular 3,197,299 bp chromosome with a guanine-cytosine (GC) content of 44.6% without plasmids. Importantly, the genome is devoid of the antimicrobial resistance gene, satisfying the minimum safety requirement for probiotics.

## ANNOUNCEMENT

A few probiotic strains of *Lactiplantibacillus plantarum* are known to inhibit oral pathogens, such as Porphyromonas gingivalis, Prevotella intermedia, and Streptococcus mutans (1–4). To isolate a potential oral probiotic strain, we spread human tongue coating biospecimens obtained from healthy donors from South Korea on de Man, Rogosa, and Sharpe (MRS) agar and identified *L. plantarum* DM083 via complete 16S rRNA sequencing in February of 2022.

For the whole-genome sequencing, DM083 was anaerobically cultivated in MRS broth at 37°C for 24 h, and genomic DNA (gDNA) was extracted using a Maxwell 16 DNA purification kit (Promega Corp., USA). For the long-read sequencing, gDNA (3 μg) was sheared to ~40 kb using a Megaruptor 3 (Diagenode, Inc., USA) and was purified using AMPure PB (PacBio, Inc., USA). The sequencing library was constructed using the SMRTbell Express Template Prep Kit 2.0 (PacBio, Inc., USA). A total of 80,239 subreads with an average length of 9,101 bp and an *N*_50_ value of 11,085 bp were obtained using the Sequel system (PacBio, Inc., USA). The Microbial Assembly protocol in SMRT Link v10.1.0.119588 yielded a single circular *oriC*-rotated genome assembly after read quality control, error correction, adapter filtering, circularity checking, and overlap trimming processes (5). For the short-read sequencing, gDNA (100 ng) was sheared using Adaptive Focused Acoustic technology (Covaris, Inc., USA), and an approximately 350 bp sequencing library was prepared using the TruSeq Nano DNA High Throughput Library Prep Kit (Illumina, Inc., USA). Sequencing was carried out on the HiSeq X Ten platform (Illumina, Inc., USA) and yielded 15,209,970 quality-filtered 2 × 151 bp reads, in which ≥90% of the bases had a Phred quality score over 30. After adapter trimming using Trimmomatic v0.38 (6), the short-reads were mapped to the long-read assembly with BWA-MEM v0.7.17 (7). Final correction of the sequence assembly with Pilon (v1.21) (8) three times with a minDepth of 0.01 yielded a 3,197,299 bp chromosome (Table 1).

**TABLE 1:** Summary of assembly and annotation statistics for *L. plantarum* DM083

Genetic element	Length (bp)	GC (%)	No. of coding seq.	No. of rRNAs	No. of tRNAs	Sequencing depth	GenBank accession no.
Chromosome	3,197,299	44.60	2,945	16	71	202.9	CP099962

An average nucleotide identity analysis using OrthoANI (9) resulted in a 99.996% sequence similarity with *L. plantarum* LM1004 (GenBank: NZ_CP025988.1). The genome sequence was annotated using the NCBI Prokaryotic Genome Annotation Pipeline v6.1 (10), which identified 2,945 protein-coding genes, 16 rRNA genes, and 71 tRNA genes. Genome annotation was also performed using Prokka v1.14.6 (11), InterProScan v5.30-69.0 (12), and EggNOG DB v4.5 (13), and these illustrated that the highest prevalence of the genes were for carbohydrate metabolism (8.8%), followed by transcription (8.3%) and amino acid metabolism (6.8%). ResFinder v4.1 (14) and the Comprehensive Antibiotic Resistance Database v3.2.3 (15) revealed that DM083 lacks antimicrobial resistance genes, which is one of the most important requirements for strain safety. The default parameters were used with all software, unless otherwise noted.

### Data availability.

The accession numbers of the genome sequence and raw sequencing reads for DM083 are CP099962 (GenBank), PRJNA853143 (BioProject), SAMN29361992 (Biosample), SRR19880474 (SRA), and SRR19880475 (SRA).
